# Nanostructures in Orthopedics: Advancing Diagnostics, Targeted Therapies, and Tissue Regeneration

**DOI:** 10.3390/ma17246162

**Published:** 2024-12-17

**Authors:** Wiktoria Frączek, Andrzej Kotela, Ireneusz Kotela, Marta Grodzik

**Affiliations:** 1 Department of Nanobiotechnology, Institute of Biology, Warsaw University of Life Sciences (WULS-SGGW), 02-787 Warsaw, Poland; 2 Faculty of Medicine, Collegium Medicum, Cardinal Stefan Wyszyński University, 01-938 Warsaw, Poland; 3 National Medical Institute of the Ministry of the Interior and Administration, 02-507 Warsaw, Poland; 4 Collegium Medicum, Jan Kochanowski University in Kielce, 25-369 Kielce, Poland

**Keywords:** nanotechnology, nanomedicine, orthopedics, diagnostics, arthroplasty, osteogenesis, chondrogenesis

## Abstract

Nanotechnology, delving into the realm of nanometric structures, stands as a transformative force in orthopedics, reshaping diagnostics, and numerous regenerative interventions. Commencing with diagnostics, this scientific discipline empowers accurate analyses of various diseases and implant stability, heralding an era of unparalleled precision. Acting as carriers for medications, nanomaterials introduce novel therapeutic possibilities, propelling the field towards more targeted and effective treatments. In arthroplasty, nanostructural modifications to implant surfaces not only enhance mechanical properties but also promote superior osteointegration and durability. Simultaneously, nanotechnology propels tissue regeneration, with nanostructured dressings emerging as pivotal elements in accelerating wound healing. As we navigate the frontiers of nanotechnology, ongoing research illuminates promising avenues for further advancements, assuring a future where orthopedic practices are not only personalized but also highly efficient, promising a captivating journey through groundbreaking innovations and tailored patient care.

## 1. Introduction

**Figure 1:**
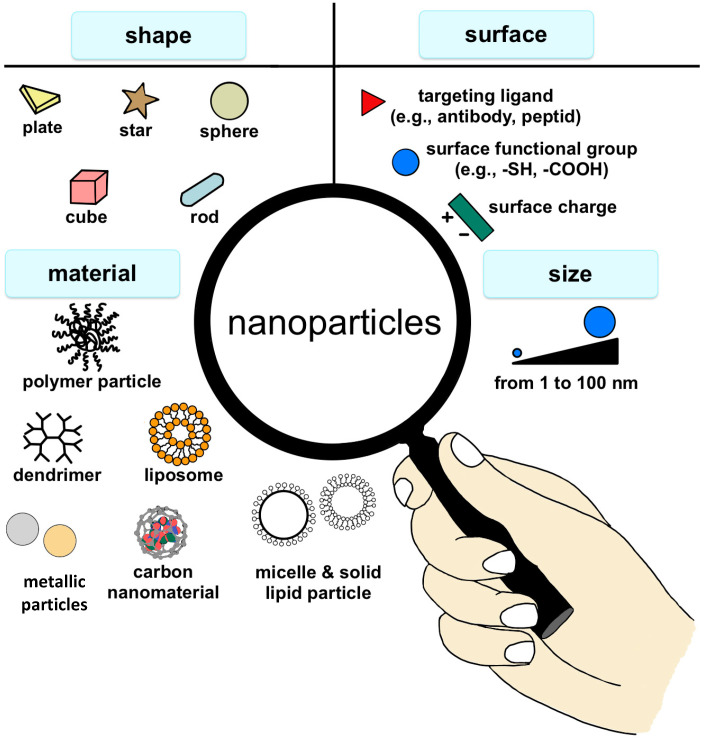
The most important factors determining nanoparticle traits along with examples of some features. Adapted from Florensa et al., 2022 [10].

Currently, advances in nanotechnology find multifaceted applications within the realm of biotechnology [1], environmental research [2,3], molecular biological investigations [4], and medical fields [5,6,7]. The capacity to exercise dominion at atomic, molecular, and supramolecular echelons inaugurates a paradigm shift in the characteristics of engendered structures and components. This is of particular significance for biological systems and their concomitant pathophysiological phenomena [8]. Nanoparticles offer an unparalleled spectrum of shapes, compositions, and sizes ranging from 1 to 100 nanometers. As we embark on this scientific journey, it is imperative to acknowledge their dynamic nature, epitomized by intricate surface modifications and a myriad of material choices. Illustrative depictions (Figure 1) unveil the intricate tapestry of nanoparticles, each possessing the potential to redefine orthopedic paradigms. From elegant spheres to intricate nanorods, these nanostructures represent a new era in biomedical innovation, poised to offer tailored solutions to the intricate challenges of musculoskeletal healing [9]. 

An interdisciplinary understanding of this phenomenon not only intimates potential trajectories for future development, but also posits itself as a catalyst for substantial improvement in the comfort experienced by both patients and medical personnel [11]. During this narrative, our objective is to explore their transformative prowess, nestled at the nexus of groundbreaking discovery and therapeutic efficacy, thus enriching our understanding of their profound impact on orthopedic practice.

## 2. Classification of Nanomaterials

Nanomaterials are broadly categorized into three main groups: organic, inorganic, and carbon-based. Each group exhibits distinct properties that make them indispensable in biomedicine, particularly in areas like orthopedic applications, cancer therapy, and targeted drug delivery. Their unique characteristics enable groundbreaking advancements in tissue engineering and regenerative medicine [12,13].

Organic nanomaterials are valued for their biocompatibility and biodegradability, making them safe for the human body and environmentally friendly. Composed of proteins, lipids, polymers, and carbohydrates, they are widely used in drug delivery systems, biosensing, and bioimaging. In orthopedics, polymer-based nanomaterials such as chitosan and alginate play a crucial role [14]. Alginate, often used to produce hydrogels, is compatible with other polymers and responsive to pH changes, providing controlled therapeutic effects [15,16]. Lipid-based nanoparticles, such as liposomes, can encapsulate both hydrophilic and hydrophobic molecules, allowing for targeted and efficient drug delivery [17]. This makes them particularly effective in treating degenerative bone diseases and inflammatory conditions [18].

Inorganic nanomaterials stand out for their exceptional physicochemical properties, including resistance to destabilizing factors, unique optical, magnetic, and electrical characteristics, and outstanding mechanical strength [12]. In biomedicine, particularly in orthopedics, they are employed in drug delivery systems and bone regeneration [19,20]. An interesting part of this group are semiconductor nanoparticles like zirconium dioxide, which are highly valued for their biocompatibility, compression resistance, and antimicrobial activity, making them ideal for supporting tissue repair [21]. Similarly, ceramic nanomaterials, including hydroxyapatite and tricalcium phosphate, mimic the natural components of bone, making them perfect for implants and bone tissue engineering. Their stability in varying environmental conditions also protects entrapped biomolecules such as proteins and enzymes from degradation [22,23]. Magnetic nanoparticles (MNPs), also belonging to the inorganic nanomaterials group, hold a prominent place in biomedicine. Their superparamagnetic properties make them indispensable in MRI imaging, targeted drug delivery, and therapies focused on bone tissues. These applications enhance both the diagnosis and treatment of orthopedic disorders [24,25,26].

Carbon-based nanomaterials, in turn, are exceptional due to their high chemical stability, electrical and thermal conductivity, and interaction with cellular structures [27]. Graphene nanoparticles, in particular, are gaining attention in orthopedic engineering. With their large surface area and ease of functionalization, graphene-based materials excel in drug delivery and tissue regeneration [28,29]. Hybrid structures combining graphene with inorganic nanomaterials exhibit synergistic properties, making them highly effective in applications such as bone repair and photothermal therapy [30].

In summary, the diversity of nanomaterials and their extraordinary properties offer unparalleled opportunities in biomedicine, particularly in orthopedics. This versatility enables precise tailoring of materials to meet specific clinical needs, paving the way for innovations in diagnosis, therapy, and tissue regeneration, which will be presented in the following chapters.

## 3. Nanoprecision in Orthopedic Diagnostics and Imaging

In recent years, nanotechnology has taken center stage in the realm of imaging, marking a new chapter in orthopedic diagnostics, therapy, and research [31]. The ability to scrutinize biological processes qualitatively and quantitatively at the cellular and molecular levels through advanced imaging methods holds the promise of early and targeted disease detection, along with monitoring treatment progress [32]. As nanotechnology advances, a range of innovative contrast agents, including those enriched with gold nanoparticles, has emerged for utilization in magnetic resonance imaging (MRI) studies [33]. These nanoparticles undergo specific modifications to selectively accumulate in regions abundant in calcium ions within bone tissues. Pioneering studies of Surender and colleagues show that surface-modified gold nanoparticles, emitting europium and enriched on surfaces rich in calcium ions, exhibit significant potential in precisely labeling microfractures within bones. This groundbreaking technique enables the identification of bone masses, facilitating the early detection of potential fracture sites [34]. 

Furthermore, the incorporation of gold nanoparticles may lead to a reduction in the concentration of contrast agents required to achieve the intended effect, which contributes to a decrease in probe toxicity [31]. However, gold nanoparticles find broader utility in orthopedic diagnostics. They have also been used in the development of a biochip designed for the efficient, fast, and precise detection of the osteoporosis-related protein osteoprotegerin. Current research indicates that the application of this technology provides remarkably precise information regarding bone damage [8]. 

In current research, intensive efforts are being directed towards harnessing the potential of modern nanotechnology to diagnose abnormalities not only within bone tissue but also within cartilage. Jin et al. have pioneered an innovative nanosensor for nitric oxide (NO), serving as a biomarker for degenerative joint diseases, enabling non-invasive and real-time monitoring of their progression. This process involves encapsulating molecules capable of detecting NO (specifically, 4-amino-5-methylamino-2′, 7′-difluorofluorescein) within biodegradable nanocarriers made of poly(lactic-co-glycolic acid). In vitro studies revealed a positive correlation between the fluorescence intensity and changes in NO concentration in chondrocytes. Furthermore, in vivo experiments confirmed the effectiveness of the sensor in quantitatively determining NO levels in the joint fluid of a rat model of degenerative joint disease [35]. This study highlights promising prospects for the use of nanotechnology in the monitoring and diagnosis of joint diseases, ushering in new horizons in this field of medicine.

Nanotechnology achievements extend beyond diagnostics, also playing a pivotal role in therapy monitoring and providing valuable insights into treatment progress. By elevating the precision and accuracy of employed sensors, nanotechnology proves indispensable in the real-time monitoring of implant force and stability. Cutting-edge technologies, such as innovative piezoresistive stress sensors integrated into prosthetics, furnish instantaneous data on implant loading. Additionally, advancements include the application of nanocrystalline silicone coatings on flexible nanocomposite substrates, detecting stress and enhancing the efficacy of implant monitoring [36]. A forward-looking strategy suggests the establishment of a sensor network for a comprehensive three-dimensional representation of the success of therapeutic interventions [37]. Another exciting avenue involves the incorporation of carbon nanotubes into titanium coatings, which offers a meticulous assessment of bone formation. This approach relies on gauging resistance in implant-developed tissues, discerning conductivity variations among bone hydroxyapatite, scar tissue, and microorganisms, ultimately enhancing our understanding of osteogenesis [38,39]. In the realm of in vivo sensors, the groundbreaking work by the Lajnef research team has triumphed over the challenges of prolonged power supply. They achieved this through ultralow-power piezoelectric strain gauges, allowing continuous sensor operation. Future endeavors aim to facilitate sensor data transmission on bone tissue development through RFID and Bluetooth technology, promising a substantial leap in healthcare information utilization [38]. 

The variety of nanostructures and their modifications are highlighted in Table 1. 

## 4. Nanoparticles Revolutionizing Drug Delivery for Orthopedic Applications

Nanotechnology, an interdisciplinary field of science, is revolutionizing therapies by enabling precise drug delivery, with applications extending to orthopedics [40]. Compared to traditionally used pharmaceuticals, drug delivery systems based on nanotechnology offer numerous advantages. One significant advantage stems from the high surface-to-volume ratio of nanoparticles, next allowing for efficient drug loading, more targeted delivery, controlled dissolution rates, enhanced drug bioavailability, and even the minimization of side effects [41,42]. In vivo studies have confirmed the potential of gold nanoparticles in targeted iontophoresis for treating tendon disorders, demonstrating their effectiveness in enhancing anti-inflammatory agents [40,43]. Another therapeutic approach involves the use of diamond nanoparticles as a carrier platform for BMP family proteins (bone morphogenetic protein), facilitating localized accumulation, proliferation, and differentiation of bone cells [44]. 

A novel approach was undertaken in the development of a controlled drug release system also aimed at addressing osteoporotic local defects. This goal can be achieved by utilizing simvastatin-loaded poly(N-isopropylacrylamide) brush-modified mesoporous hydroxyapatite nanoparticles [45]. This system ensured sustained release of simvastatin to counteract osteoporosis, while concurrently leveraging hydroxyapatite nanoparticles to promote osteogenesis. Additionally, the potential of alendronate-conjugated nanodiamonds was also explored as a bone-targeted delivery system for treating osteoporosis, aiming to achieve a synergistic effect. The research highlights specific accumulation in bone tissue, strong affinity to hydroxyapatite, positive synergistic effects on alkaline phosphatase activity, and in vivo bone targeting capabilities as promising attributes of alendronate-conjugated nanodiamonds [46]. Analyses of nanotechnology applications in drug delivery are also crucial during surgical procedures. A nanofilm composed of biodegradable polypeptide and cefazolin has shown potential in reducing infection risks and promoting bone growth after implantation surgeries related to joint implants. Notably, this system influences the pharmacokinetics of antibiotic release, allowing concentrated action during a critical period after implantation [11,47].

However, the therapeutic use of nanotechnology is not limited to providing compounds that promote bone cell proliferation and prevent infections. This field of study also plays a pivotal role in anticancer therapy, especially in bone cancer. Ongoing research encompasses the creation of a biocomposite of collagen hydroxyapatite with nanomagnesium for bone transplantation, and nanocarriers loaded with cisplatin showing efficacy against osteosarcoma cells while minimizing the accumulation of drugs in the kidneys and side effects [47,48]. Another example is doxorubicin, an immunogenic cell death-activating drug, which is also of significant importance in bone cancer therapy. Innovative nanotechnology-based approaches introduce strategies for the delivery of doxorubicin, such as phosphate-doped selenium. This pH-dependent carrier enables specific doxorubicin release in tumor areas, enhancing drug absorption by cancer cells. Moreover, released selenium ions reduce reactive oxygen species in cancer cells and decrease ATP-binding cassette transporter expression, contributing to overcoming multidrug resistance. This comprehensive nanotechnology-based approach directs the effective action of doxorubicin towards the tumor area, minimizing potential side effects. [49,50]. Drawing upon existing knowledge has facilitated the exploration aimed at developing an innovative gel formulation composed of hyaluronic acid crosslinked with cisplatin and loaded with doxorubicin, for effective osteosarcoma treatment. This sophisticated investigation has led to the extension of substance circulation time due to the stability of the nano-gel, a feature unparalleled in free drugs. Synergistic apoptotic effects induced by doxorubicin and cisplatin were observed upon proper tumor accumulation, underscoring significant potential for osteosarcoma chemotherapy [51]. Alternative applications of nanoparticle-based drug delivery targeting bone tumors have been explored extensively. In a separate study by Au et al., pH-responsive nanoscale metal–organic frameworks were developed for delivering calcium zoledronate in the treatment of bone metastases from cancer [52]. Folate, a targeting ligand, was incorporated to facilitate tumor uptake by the nanoparticles [53]. The authors demonstrated an 80 to 85% increase in the direct anticancer activity of zoledronate in vivo compared to the free drug, attributed to improved tumor targeting and extended drug release kinetics [52]. Similarly, an intelligent drug delivery system composed of functionalized graphene oxide conjugated with folate, polyethylene glycol, and green indocyanine photothermal and photodynamic catalyst was developed for delivering MutT homolog 1 inhibitor (protects DNA from oxidative damage) and doxorubicin. This nanoparticle delivery system, responsive to the low pH environment in the tumor and possessing photothermal and photodynamic transformation capabilities, exhibited combined chemo-photodynamic effects to inhibit osteosarcoma growth [52].

Data on the utilization of nanotechnology for drug delivery suggest that an interdisciplinary approach can enhance therapy precision, limit bacterial growth, reduce infection risks, and support the field of orthopedic oncology (Table 2). This holds paramount significance for the continued development of orthopedic medicine, contributing to improved therapy efficacy and enhanced quality of life for patients [11].

## 5. Enhancing Arthroplasty with Nanotechnology Integration

The hip and knee joints, also known as synovial joints, are characterized by smooth articular surfaces that facilitate natural, smooth, and painless bone movement. However, the susceptibility of these joints to wear and tear can lead to pain and subsequently the onset of various conditions [54]. To restore mobility and relieve discomfort, arthroplasty procedures are used, which involve replacing the damaged joint with an artificial device called a prosthesis. The benefits associated with such a surgical procedure persist for more than two decades after the initial surgery, and are reproducible and cost-effective [55,56]. Nevertheless, increasing attention is being paid to limitations that negatively affect patient and medical staff comfort [57]. Among the factors associated with the incidence of early and rapid or chronic (occurring over time) failures of total joint replacement, the surgical technique, polyethylene wear, loosening, instability, or dislocation of implants are pointed out [58,59]. Current research in joint arthroplasty focuses on several key areas to enhance the effectiveness and longevity of implants. Scientists explore, among others, advanced biomaterials for long-term implant functionality, with a goal of prosthetic durability that exceeds 30 years. In addition, attention is being directed towards developing effective interfaces between the implant and bone, known as fixation, to improve the osseointegration of the prosthesis. Research efforts also concentrate on preventing wear and contaminants on the bearing surface [60,61].

**Figure 2:**
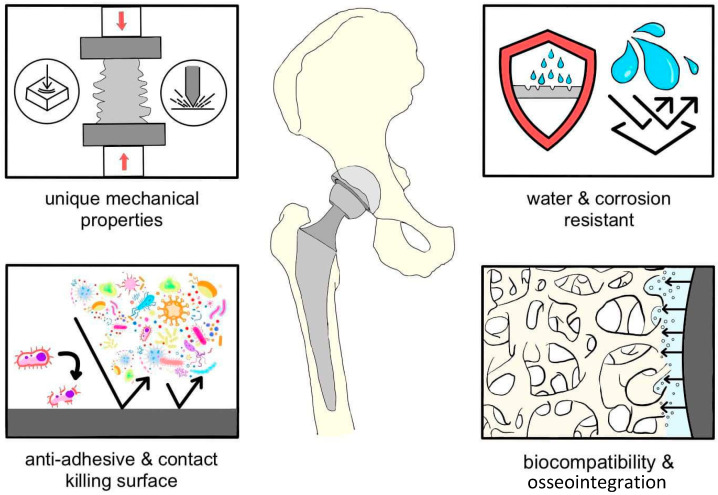
Key requirements of an orthopedic implant. Adapted from Chen, 2022 [66].

In the context of implant integration into bone, new and improved methods of functionalizing implant surfaces by modifying chemical and mechanical properties are being explored. It has been suggested that biomimetic surfaces increase cell interactions in the biological environment, ultimately improving the effectiveness of the implant [62]. It is worth bearing in mind that the introduction of synthetic materials into the body may trigger inflammatory reactions, limiting device integration. For this reason, protective coatings are proposed to control foreign body reactions, maintain long-term mechanical stability, and prevent adverse reactions [63]. One common adverse reaction after joint replacement surgery is the release of cobalt and chromium ions into the bloodstream on metal-to-metal surfaces, posing a potential health risk [64,65]. Ceramic surfaces have been suggested as a solution, although this requires advanced design due to the thickness of the implant. Clinical practice increasingly emphasizes the need for arthroplastic implants to meet a set of critical properties to ensure efficacy and patient safety (Figure 2). The introduction of new solutions requires a balanced approach, considering both benefits and potential risks, to effectively contribute to advances in orthopedic surgery [60].

Nanotechnology, owing to its advanced manipulation capabilities at the nanometric scale, emerges as a promising field poised to revolutionize endoprosthetics. Precision tuning of implant structures using nanotechnology holds promise for enhancing their durability, biocompatibility, and adaptability to changing conditions within the patient’s body. Addressing the design challenges, nanotechnology can introduce innovative solutions, minimizing limitations associated with traditional methods of endoprosthetics. It is suggested that the modification of implant surfaces using contemporary nanotechnology achievements may enhance interactions between bone and endoprosthesis, directly influencing osseointegration and implant durability [37]. 

A particularly promising prospect in the context of orthopedic transplants are manipulations in the proportions of the basic components of biological bone. This area has been intensively researched, with monomers and nanocomposites of nanomaterials finding applications in bone tissue engineering, aiming to stimulate osteoblast activity, improve osseointegration, and address bone-related disorders [67]. In the practical implications of nanotechnology in knee joint endoprosthetics, nano-sized structures such as nanofibers, nanotubes, nanocubes, quantum dots, nanorods, nanoflowers, and metal–organic frameworks are extensively studied [68,69,70,71,72,73,74]. These investigations focus on analyzing the beneficial surface characteristics of nanoscale components, supporting precise protein relationships, promoting osteoblast growth, and stimulating the development and movement of osteoblasts for more efficient bone growth compared to traditional tools [75,76]. Nanotechnology development has brought forth a wealth of nanophase components with particle sizes of 100 nm, encompassing metals, composites, and polymers with exceptional surface properties. The extraordinary properties of these materials, such as enhanced osseointegration capability and stimulation of new bone development, constitute the focal point of scientific analysis in orthopedic implantology [75]. Particle size reduction, such as titanium from 4500 nm to 200 nm, can significantly enhance cell propagation, as confirmed by studies by Estrin et al. [77]. Nanophase components, characterized by grain boundary density, exploit differences in atomic structure, making polycrystalline nanomaterials simultaneously brittle and ductile [78,79]. The flexible nature of materials at the nanoscale poses challenges, especially in advanced construction applications. Moreover, the application of intense plastic deformation techniques has shown the ability to improve the mechanical properties and biocompatibility of implants by reducing coarse metal fragments to the nanoscale [80,81]. Similar approaches are anticipated in the case of ultra-high-molecular-weight polyethylene, a highly biocompatible material prone to potential fractures, where reinforcement with carbon nanotubes is of particular interest [82]. 

Also, surface nanostructure modifications of implants can increase their resistance to static and dynamic fatigue, improve functionality, and extend implant durability [37]. The versatility of coatings applied to prostheses stems, among other factors, from the diversity of nanomaterials that can be utilized for this purpose. Surface optimization of the implant can promote angiogenesis, osteoinduction, osteoconduction, and osteogenesis, as well as possess antibacterial properties. Furthermore, the ability to combine nanomaterials allows for the creation of multi-component coatings, which amplify benefits, enhancing the effectiveness of endoprostheses while minimizing the risk of infection. Among the most widely used materials that could revolutionize arthroplasty is tantalum, attracting attention both as an implant component and as a surface coating. Tantalum is characterized by its biocompatibility and high corrosion resistance, making it an attractive choice. Its ability to promote bone integration and osteoinduction, key parameters affecting secondary implant stability, has led to increased interest in this metal in orthopedics [83,84,85]. The same attention is also being paid to the use of titanium in orthopedics, which can come in a variety of sizes and shapes [86]. Titanium dioxide nanotubes are specifically designed to enhance the bioactivity and osteointegration of implants. Moreover, their antibacterial properties, enhanced by antibiotic compounds such as vancomycin and gentamicin, offer new possibilities for controlling infection around implants [87,88,89]. The use of these nanotubes further stimulates osteogenic stem cell differentiation by increasing adhesion to osteoblasts, thus facilitating better integration into bone tissue [90].

In conclusion, the integration of nanotechnology in joint arthroplasty represents a significant advancement, with the potential to address current limitations and revolutionize orthopedic practice. Through precise engineering of implant structures and surface modifications at the nanoscale, researchers aim to enhance implant efficacy, durability, and patient outcomes. As research in this field continues to progress, the adoption of nanotechnology holds promise for optimizing orthopedic implants and improving the lives of patients undergoing joint replacement surgeries [60] (Table 3).

## 6. Bone Tissue Engineering and Osteogenic Potential of Nanomaterial

Orthopedic traumatology analyses focus on refining the osteointegration of implants and promoting proper bone growth [11]. However, bone regeneration following injury or surgical intervention remains a significant medical challenge. Regeneration necessitates cells capable of proliferation and differentiation into bone tissue [91]. This process is intricate, involving numerous skeletal cells—osteoblasts, osteoclasts—as well as numerous immune cells regulating bone formation and resorption [92].

**Figure 3:**
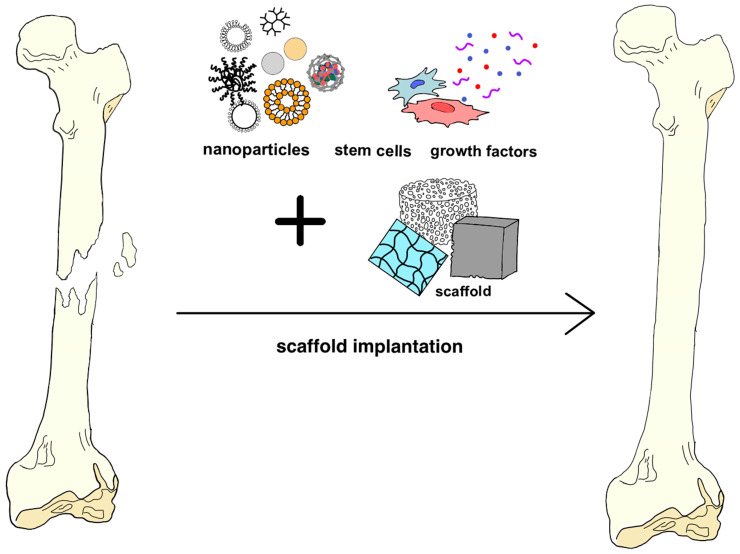
Schematic representation of optimal scaffolds for bone tissue regeneration containing nanoparticles, stem cells, and growth factors. Adapted from Bauso et al., 2023 [67].

Within the realm of regenerative medicine, a prominent research objective is the development of bioactive scaffolds supporting bone regeneration, reducing healing time, and restoring full tissue functionality. Studies demonstrate that nanofiber scaffolds facilitate cell migration and growth during bone healing, confirming their ability to stimulate osteogenesis [44,93,94,95]. Advances in nanotechnology also open avenues for innovative approaches to bone reconstruction, eliminating the need for conventional grafts. Synthetic materials mimicking the native bone structure are employed in this context [96,97]. Recent studies have shown that nanoparticles (NPs) could influence bone regeneration by enhancing cellular signaling, proliferation, and osteogenic differentiation. They can be produced from various materials and are typically combined with different matrices to develop nanocomposite scaffolds [98,99,100] (Figure 3).

Among natural biopolymers, collagen type I (Col) and fibrin are noteworthy candidates for use in bone engineering when combined with nanomaterials. Col, as the organic component of the bone matrix, is increasingly becoming a vital element in the development of new implants. However, certain limitations such as poor mechanical properties, weak osteoinductivity, and rapid degradation continue to hinder its widespread application [101]. To address these shortcomings, numerous attempts have been made to enhance conventional bone tissue repair implants using collagen type I nanoparticles. For instance, collagen–apatite has been shown to promote osteoblast proliferation and differentiation while improving vascularization at the defect site in vivo. Similarly, it has been observed that collagen–hydrogel nanocomposites enhance bone mineralization [67,102]. It has been reported that a biocompatible synthetic polymer consisting of HA and PLGA significantly improved osteoblast adhesion and proliferation of human osteosarcoma cells after just 7 days of in vitro culture [103]. Fibrin, well known in the orthopedic field for its role in the coagulation process, provides support for extracellular matrix synthesis. As a result, it promotes cell adhesion and proliferation during wound healing and bone growth. Its precursors can be obtained from the patient’s blood, allowing for the development of purely autologous and cost-effective scaffolds that can be controlled during the manufacturing process by adjusting the concentration of components. The impact of a fibrinogen coating on the surface of biphasic calcium phosphate under in vitro conditions has been studied, with significant improvements reported in the adhesion and proliferation of human mesenchymal stem cells [104,105]. Others have also noted that fibrinogen deposition in scaffolds enhances bone regeneration in vivo. However, due to its rapid degradation and poor mechanical properties, many researchers have combined fibrin with nanotechnology to overcome these limitations [106,107]. Periyathambi et al. developed innovative magnetic fibrin nanoparticles capable of enhancing cell viability and activity in vitro [108].

Another excellent example of supporting tissue regeneration using polymers is the utilization of polylactide (PLA). Several studies have demonstrated the ability of PLA nanofibers, either used alone or in combination with other molecules, to promote cell growth and osteogenic differentiation [109,110]. One such development includes a scaffold composed of nanocomposite PLA fibers with graphene oxide (GO) and nanohydroxyapatite. The resulting nanocomposite scaffold exhibited high biocompatibility, tensile strength, and excellent cell proliferation [111]. Other carbon-based nanomaterials also exhibit properties highly valued in bone tissue engineering. Diamond nanoparticles (NDs) possess a high surface-to-volume ratio, as well as high surface reactivity and extreme hardness, along with biocompatibility. They can be functionalized or oxidized to overcome their hydrophilicity and dispersion limitations [112]. Particularly, ND films have been found to be promising for tissue engineering applications due to their ability to promote adhesion, proliferation, and osteoblast differentiation in vitro. Moreover, the incorporation of NDs into conventional scaffolds such as PLA and poly(glycolide-co-L-lactide) (PGLA) has been shown to enhance their mechanical and biological properties [113,114].

However, this is not the end of the possibilities for utilizing polymeric scaffolds and nanomaterials. Also, hybrid nanocomposites composed of bisphosphonated hyaluronan and calcium phosphate nanoparticles have been successfully engineered with reversible, non-covalent bonds, showcasing self-healing properties, a significant stride towards regenerative capability. This composite, exhibiting excellent biocompatibility, interacts effectively with bone implants and displays strong adhesion to enamel and hydroxyapatite surfaces [115]. Similarly, Chang et al. developed uniform apatite nanorods based on carbonates, enhancing cellular attachment and mineralization by incorporating ferulic acid through heparin regulation [116]. An alternative approach to bone regeneration involves the utilization of nano-hydroxyapatite. This material, aside from its confirmed biocompatibility, also exhibits osteoconductivity, serving as a thriving substitute for bone tissue. It has been observed that tantalum scaffolds coated with nano-hydroxyapatite enhance the formation of new bone as early as 2 weeks post-implantation of rat calvarial bone compared to scaffolds coated with micron-sized hydroxyapatite [117].

It also must be noted that stem cells play a pivotal role in bone regeneration, and studies reveal that calcium-based materials can burden these cells [118,119]. The interaction between nanostructures and cells contributes to bone healing [120]. An example of this is constituted by calcium phosphate bone cement, combined with mesenchymal stem cells from bone marrow, condensed into alginate–chitosan microcapsules. They function as protective carriers for the mesenchymal stem cells, shielding them from potential adverse environmental conditions or immune responses. The encapsulation process provides a controlled environment, enhancing the survival and retention of the stem cells at the targeted site of application, which is crucial for their therapeutic effectiveness. Calcium phosphate-based nanomaterials exhibit excellent biocompatibility, osteoinductive properties, and possess a significant surface area and porosity, making them widely applied in bone regeneration [121,122].

An incredibly significant aspect to consider when discussing bone tissue regeneration is also paying attention to osteoimmunology, defined as the study of communication between the skeletal and immune systems [123,124]. The undeniable integrity of both systems is reflected in aspects related to the pathogenesis and progression of musculoskeletal pathologies. Osteoclasts, osteoblasts, and numerous immune cells interact with each other and the surrounding microenvironment, determining and regulating processes such as regeneration [125]. This environment can be modulated to achieve tangible benefits, including using nanoparticles. Particularly crucial in this context appear to be actions targeting macrophages—critical regulators of bone regeneration—as phagocytic cells preferentially uptake nanoparticles [92,126]. The role of M1 macrophages encompasses the regulation of intracellular pathogen levels and subsequent release of pro-inflammatory cytokines, while activation of the M2 phenotype primarily triggers anti-inflammatory responses and subsequent tissue repair [127]. Recent findings, however, suggest that the presence of M1 macrophages enhances osteogenesis whereas excessive transition to the M2 phenotype leads to fibrotic tissue healing [128,129]. Thus, effective modulation of the M1 to M2 polarization at the appropriate timing is essential. Therefore, it is believed that utilizing nanobiotechnology for targeted macrophage polarization could significantly improve bone regeneration. Examples include gold nanoparticles, titanium dioxide, and cerium oxide, which have shown positive effects on M2 macrophage polarization. Furthermore, it has been demonstrated that not only the chemical properties of the materials used but also their physical characteristics, such as surface structure and pore size, influence the inflammatory response and the release of pro-osteogenic factors by macrophages. This, in turn, affects their spreading, cellular morphology, adhesion capability, and may impact bone metabolism [130,131]. Increasing the pore size of nanoparticles leads to the development and proliferation of macrophages with distinct anti-inflammatory properties, resulting in reduced expression of surface markers characteristic of the M1 phenotype. Additionally, nanoparticles with rough surfaces also influence macrophage activation and cytokine release. Studies have shown that titanium (Ti) with a smooth surface may stimulate M1 phenotype activation and the secretion of inflammatory cytokines, whereas Ti with a rough and hydrophilic surface promotes anti-inflammatory macrophage polarization and the release of some cytokines such as inteleukin-4 and interleukin-10 [132]. An alternative strategy for fostering M2 polarization involves altering the surface composition of NPs, such as through the incorporation of bioactive molecules [133,134]. For instance, peptides like Cys-Leu-Pro-Phe-Phe-Asp, the arginyl–glycyl–aspartic acid peptide, and interleukin-4 have been successfully conjugated onto gold NP surfaces to elicit potent anti-inflammatory effects [135,136,137]. CeO2 NPs, conversely, have been surface-coated with hydroxyapatite to promote M2 polarization [138]. Previous investigations have demonstrated that modifying the surface of hydroxyapatite nanorods with chitosan diminishes macrophage activation while enhancing osteoblast proliferation [92]. Additionally, in a study, microcrystalline bioactive glass scaffolds incorporating varying levels of ZnO were developed, facilitating sequential M1 to M2 macrophage polarization [139].

While only a subset of discoveries have been referenced, they collectively highlight the clear indication that the integration of nanotechnology into orthopedic traumatology and regenerative medicine holds tremendous promise for advancing bone healing and reconstruction, offering innovative solutions for enhancing tissue regeneration and functional recovery [140] (Table 4).

## 7. Nanotechnological Advancements in Cartilage Regeneration

Equally important are analyses related to the reconstruction of cartilage tissue. Cartilage is one of the most challenging tissues to treat due to its limited self-regenerative potential. In contrast to most tissues in the human body, cartilage cells are sparsely distributed, and due to their avascular, neural, and lymphatic nature, access to nutrients and circulating progenitors is highly restricted [141]. With the demographic shift towards an aging and obese population, the risk of cartilage damage in the population has significantly increased. Disease states, including arthritis, often diagnosed in clinical practice, involve a variety of challenges due to trauma, inflammatory processes, and degeneration associated precisely with aging tissue [142,143]. Symptoms of such conditions, including pain, joint dysfunction, and deformities, impact not only patients but also the healthcare system, significantly reducing the quality of life [144]. Despite existing surgical methods, cartilage regeneration by traditional methods often fails to meet clinical expectations [145]. Osteochondral tissue regeneration strategies must recover both zoned articular cartilage and subchondral bone, restoring structural, biomechanical, and biochemical characteristics consistent with native osteochondral tissue [146]. In response to these challenges, cartilage and subchondral bone repair remains an ongoing challenge in orthopedics [147]. 

Nanomedicine, as a field focusing on the application of nanotechnology in medicine, also emerges as a promising tool to enhance therapeutic outcomes in this area of orthopedics related to the regeneration of cartilage tissue. Preclinical studies suggest the potential of nanomaterials in creating biocompatible scaffolds for mesenchymal stem cells (MSCs), positively influencing the proliferation of chondrocytes. Scaffolds made of nanofibers such as polycaprolactone, gelatin, polyethersulfone, and hyaluronic acid analogs demonstrate the ability to influence the differentiation of MSCs towards the chondrogenic lineage [148,149,150,151,152,153]. The utilization of hydrogel scaffolds can be optimized by integrating nanomaterials into their matrix [154]. Organic/polymeric and inorganic nanoparticles such as hydroxyapatite, clay, graphene, and metallic nanoparticles serve as potential fillers to reinforce hydrogel matrices and even confer novel functionalities upon them [155]. This approach is exemplified by the work of Chen et al., who combined superparamagnetic iron oxide particles with cellulose nanocrystals and silk fibroin to prepare magnetic hydrogels. Degradation and regeneration processes were evaluated using multiparametric magnetic resonance analysis [156]. In a separate study, magnetic manipulation was employed to mechanically stimulate MSCs to promote cartilage differentiation. The experiment utilized gelatin-based β-magnetic nano-hydrogels prepared with cyclodextrins and Fe_3_O_4_ as the magnetic material. The cell activity results indicate the biocompatibility of the magnetic hydrogel, where both the material and MSCs effectively repaired cartilage under pulsed electromagnetic field stimulation in rabbits. After 12 weeks, the regenerated tissue filled the defect and exhibited characteristics of natural cartilage, as confirmed by histological analysis [157]. Additionally, research on hydrogels, such as poly(ethylene glycol) diacrylate (PEGDA) hydrogel, shows promising results in the regeneration of cartilage and joints. The integration of chondroitin sulfate adhesive with the hydrogel promotes the adhesion of the implant to cartilage and bone tissue, consequently improving joint function [158]. Although most analyses focus on preclinical studies, advanced pilot studies, such as the assessment of biomimetic osteochondral scaffolding with a nanostructured collagen–hydroxyapatite complex, show substantial regeneration of cartilage after a two-year analysis [153]. 

**Figure 4:**
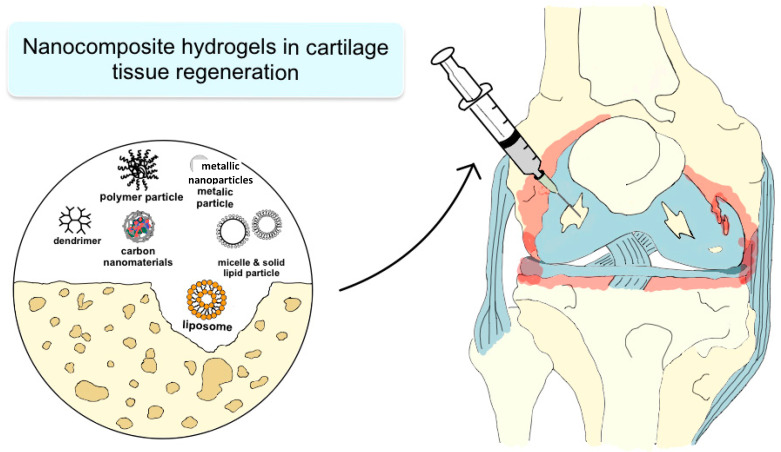
Schematic representation of cartilage tissue regeneration by injections with hydrocolloids containing a variety of nanocomposites. Adapted from Lagneau et al., 2023 [159].

Another approach with a potentially significant impact on cartilage tissue regeneration is the direct application of hydrogels to the joint (Figure 4).

This presents a perspective for substantial and enduring improvement in joint mobility, as well as alleviation of pain and discomfort [160]. In contrast to pre-formed scaffold materials, injectable hydrogels have demonstrated greater suitability for osteochondral tissue engineering due to their ease of application and enhanced compatibility with patient tissues. This approach is recognized for its reduced invasiveness and potential for targeted therapy development. Injectable hydrogels administered into joint spaces can be directly delivered even to large irregular or geometrically complex sites for tissue repair. Given their patient-friendly nature, they are widely employed in cartilage tissue repair [161]. Numerous reports have documented the use of injectable hydrogels in osteochondral tissue engineering [162,163]. For instance, Lee et al. utilized a copolymer matrix of polyethylene glycol–poly-L-alanine–polyaspartic acid to prepare injectable hydrogel scaffolds, incorporating cartogenin for chondrocyte induction and applying nanocomposites of double hydroxide with refined aspartic acid glyceride. Compared to a pure water gel system, the addition of the nanocomposite system enhanced cell aggregation and significantly improved the expression of mesenchymal stem cell cartilage biomarkers [164].

An innovative approach worth highlighting involves harnessing bioparticles released by resident cells and those comprising the extracellular matrix (ECM) during cartilage regeneration [165]. The intricate composition of decellularized ECM materials at the nanoscale encompasses an ultrastructural microenvironment containing embedded physicochemical and biological cues crucial for successful tissue regeneration [166]. Following the removal of cellular constituents, the cartilage ECM retains a biomolecular framework capable of stimulating chondrogenic development without provoking an adverse immunological response [167]. However, to date, few studies have utilized the acellular ECM for the synthesis of nanoparticles for chondrogenic differentiation. Saeed Zahiri et al. developed acellular ECM nanoparticles with an average size of approximately 60 nm, which have been demonstrated to promote the chondrogenic development of human primary chondrocytes in micro-mass spheroids by preserving natural ECM signals [168]. These findings suggest that acellular ECM nanoparticles may serve as valuable supplements in culture media and injectable biomaterials, particularly in cell therapies for cartilage tissue regeneration [169].

While nanotechnology in cartilage tissue regeneration has not yet achieved widespread clinical application, it shows promising results. The utilization of nanomaterials as scaffolds for engineering regenerative tissues seems to be a promising avenue, positively impacting cell adhesion, proliferation, and phenotypic selection of chondrocytes (Table 5). This perspective opens new possibilities in the field of treating cartilage and joint injuries, with the hope of improving the quality of life for patients [170].

## 8. Prevention of Nanotechnological Infection and Its Treatment 

Chirurgical interventions in orthopedics, frequently employed for treating degenerative joint diseases, have given rise to a troubling surge in infections [171]. These infections, predominantly induced by opportunistic pathogens and bacteria inherent to human skin, can manifest both pre- and post-surgery, encompassing consequences that span from nullifying therapeutic effects to necessitating subsequent surgical interventions [172,173]. The impact of infections linked to orthopedic surgery extends beyond mere healthcare costs, extending into the realm of socioeconomics [174]. Adding to the complexity is the escalating bacterial resistance resulting from the improper use of antibiotics within medical institutions and society at large. This, in turn, poses profound implications for the treatment of infections associated with orthopedic implants [175].

Even when antibiotics are administered out of medical necessity, their use can bring about unwanted secondary effects. The conventional strategy of systemic antibiotic administration, while common, is not without drawbacks. Potential inadequacies include the failure to reach the required concentrations at sites prone to infection, such as fracture locations, thereby affecting bone structural integrity. Furthermore, elevating systemic antibiotic dosages raises the risk of toxicity [176,177]. This prompts the exploration of alternative paradigms, and one promising approach involves the localized delivery of antibiotics. This not only mitigates the hazard of systemic toxicity but also optimizes concentrations at the target site, potentially enhancing therapeutic outcomes. However, concerns persist regarding the cytotoxicity associated with elevated antibiotic concentrations, underscoring the imperative for the development of stable and controlled delivery systems. In the face of increasing bacterial resistance to antibiotics, the perpetual quest for effective and innovative solutions within orthopedic surgery remains an ongoing challenge [178].

Advancements in nanotechnology have played a pivotal role in the convalescence process, particularly in the formulation of dressings, preparations, and dispersions comprising nanometric constituents [179]. This marks substantial strides in regenerative medicine, where nanoparticles endowed with antimicrobial attributes, such as silver, copper, quantum dots, and zinc oxide, have assumed pivotal roles and become subjects of intensive scrutiny within biomaterial applications [180]. Take silver nanoparticles (AgNP), for instance. They manifest an intrinsic capacity to inflict damage upon bacterial cell membranes, interact with protein sulfhydryl groups, and deactivate respiratory enzymes. These mechanistic underpinnings constitute the bedrock of their antimicrobial efficacy, portending potential efficacy in surmounting bacterial resistance [181]. Importantly, silver nanoparticles exert an impact on cellular viability without impeding the osteogenic processes indispensable for proper bone growth that encompass the implant. However, scrutiny of silver nanoparticles additionally accentuates challenges intrinsic to their stabilization and integration into biomaterials. Predilections towards aggregation and the specter of cytotoxicity stand as salient predicaments. A pioneering resolution involves the amalgamation of silver nanoparticles with polymers, affording controlled ion release while minimizing the concomitant risks linked to heightened local silver concentrations. Critical nuances in the selection of silver nanoparticle morphology are paramount in determining their antimicrobial efficacy [182]. Notably, triangular silver nanoparticles may exhibit superior efficacy in inhibiting *Escherichia coli* bacteria growth compared to their spherical or rod-shaped counterparts [183]. Notwithstanding promising prospects surrounding the application of silver nanoparticles in biomaterials, synthesis methodologies reliant on organic solvents and stabilizers provide environmental toxicity concerns. Consequently, the imperative lies in the cultivation of "green" synthesis methodologies, utilizing substances such as supercritical CO_2_, starch, glucose, or gelatin as reducers and stabilizers for silver nanoparticles [184,185].

The recent literature has underscored the merits of various graphene derivatives, including multilayer graphene, graphene oxide (GO), and reduced graphene oxide (rGO), due to their mechanical and antimicrobial properties. GO materials inherently possess antibacterial properties, conjoined with antiadhesive and contact properties. However, an imperative undertaking involves ascertaining the durability of the GO coating and discerning varied levels of bacterial susceptibility to the antibacterial action of GO [186].

Recently, the use of nanotechnology and nanoparticles to disrupt biofilms from external sources (lasers, alternative magnetic fields—AMFs) has been posited as a methodological framework. Elevated nanoparticle temperatures have been demonstrated to inflict irreversible thermal damage upon bacterial cells that reside within biofilms. In a parallel investigation, gold-loaded antibiotic spheres were harnessed to disrupt the biofilms of *Staphylococcus aureus* and *Pseudomonas aeruginosa*, with pulsed laser irradiation effecting the direct activation of antibiotic release into mature biofilms [187,188]. Magnetic nanoparticles present an alternative avenue for inducing thermal damage within biofilms, with the heating of magnetic nanoparticles (iron oxide) effectively disrupting the biofilms of Staphylococcus aureus and other microorganisms [189,190].

An alternative nanotechnological approach to dressings involves the employment of polyurethane membranes. Characterized by an architecture that features exceedingly fine fibers, these membranes facilitate the creation of products that boast a substantial surface area relative to the mass. Noteworthy attributes encompass outstanding permeability and the ability to sustain desired moisture levels, thereby fostering an optimal wound milieu—a particularly crucial consideration in orthopedics given the pervasive risk of postoperative infections [191].

Regrettably, the clinical translation of these thermal technologies remains circumscribed by the potential hazard of damaging adjacent and distal tissues. As we navigate the intricate landscape of orthopedic interventions and infectious complications, the integration of cutting-edge nanotechnological solutions holds the promise of revolutionizing treatment paradigms and overcoming persistent challenges in the realm of orthopedic surgery [179] (Table 6). 

## 9. Limitations Associated with the Clinical Application of Nanoparticles

The undeniable potential of nanotechnology in medical applications has been substantiated by research, affirming the health advantages offered by nanomedicine [192]. However, the integration of these innovations into clinical practice has been accompanied by several challenges up to this point. The main hurdle integrated with the application of nanomedicine arises from the diverse nature of nanoparticles, all of them unique in size. Furthermore, variations in the shape and dimension can trigger various physical and chemical interactions; they may disintegrate or aggregate, resulting in size fluctuations that induce toxicity [193]. Nanoparticles can also exhibit different behaviors depending on the pH of the surrounding environment, significantly influencing their stability and aggregation. To address this, surface modifications are often employed to optimize nanoparticle properties in specific pH ranges found in biological fluids or tissues, ensuring their stability, and minimizing aggregation under these conditions [194,195].

Furthermore, the ability of organisms to eliminate them or their potential accumulation within the body remains unpredictable **[193]**. A pivotal consideration involves understanding the enduring impact of diverse and specific nanomaterials on the human organism, particularly in elucidating their biodistribution and interactions with biomolecules. Surface modifications such as PEGylation can enhance biocompatibility and stability, allowing nanoparticles to evade immune detection and improve their performance in biological systems [195,196]. Also, the new approach takes into account future applications of “smart” nanoparticles, which can respond to external stimuli such as temperature, pH, or the presence of specific biomolecules. Moreover, self-degradable nanomaterials, designed to break down only after performing their function, can minimize the environmental impact while ensuring they fulfill their therapeutic or diagnostic role. Such materials, when carefully engineered, degrade safely in the body without affecting their functionality during the required time frame [197,198,199]. 

Beyond the evident patient risks, nanoparticles also harbor potential environmental toxicity and may necessitate pre-processing prior to disposal because some of them can have the potential to contaminate soil, water, or air [200]. In this regard, environmentally friendly disposal methods or degradation processes for nanomaterials are being developed to mitigate their environmental impact. This includes the use of biodegradable nanomaterials, which are designed to degrade after fulfilling their intended function in the body or environment, preventing premature breakdown [201]. Such materials ensure that their utility is not compromised before they have performed their role, yet they are safely broken down in natural systems once their purpose is completed [202].

The challenges also persist in the realm of clinical studies, where stringent requirements exert a direct influence on the temporal aspects of the incorporation of nanotechnology into clinical frameworks [11]. The intricacies extend to the mass synthesis of nanomaterials, which poses a significant obstacle. Establishing production standards holds promise for enhancing the reproducibility of studies and optimizing the efficacy of nanomaterial interventions. This is imperative, as even minor deviations from established norms can exert a substantial influence on the properties of the resulting products. Commercialization of nanomaterials, particularly within the field of orthopedics, is concerned with the delicate balance between an efficient production model and fiscal constraints because the constraint in the application of nanotechnology in medicine lies in its exorbitant expense. This dynamic can significantly impact companies’ decisions regarding substantial investments in the ongoing development of these groundbreaking technologies [11,200].

## 10. Future Prospects 

The integration of artificial intelligence (AI), machine learning (ML), and nanotechnology in orthopedics represents an emerging field of research with immense potential to revolutionize medical practices. While still in its infancy, the convergence of these technologies promises groundbreaking advancements in the diagnosis, treatment, and prevention of musculoskeletal disorders. As technological innovations continue to progress at an unprecedented pace, it is expected that this intersection will lead to highly precise and personalized therapies, dramatically improving clinical outcomes and patient care [203,204].

AI and ML can play a pivotal role in diagnostics, demonstrating exceptional accuracy in recognizing implants from radiographs, diagnosing fractures, and identifying joint diseases such as osteoarthritis. These AI-powered tools surpass traditional methods in both speed and reliability, significantly enhancing preoperative planning, postoperative care, and ongoing patient monitoring. Furthermore, AI-driven predictive models, such as convolutional neural networks (CNNs), excel at detecting subtle patterns in radiological data, forecasting disease progression, and even predicting implant behavior. When combined with real-time monitoring of implants and surrounding tissues, AI provides actionable insights that optimize treatment plans and improve patient outcomes [203]. Nanotechnology complements these advances by enhancing diagnostic imaging and drug delivery systems. For instance, quantum dots and other nanoparticles improve imaging resolution, enabling early-stage detection of pathologies at the cellular level. In parallel, nanotechnology has revolutionized drug delivery by enabling the design of nanoparticles that can target specific tissues, improving bioavailability while minimizing systemic side effects [205,206]. AI models further optimize nanoparticle design, ensuring that drugs—such as anti-inflammatory agents or growth factors—are delivered directly to the site of injury, thus enhancing the efficacy of treatments and supporting personalized medicine [207,208].

In the realm of orthopedic implants, nanotechnology and AI are fostering the development of dynamic, adaptive solutions that could redefine surgical practices. One such innovation is 4D printing, which goes beyond traditional 3D printing by incorporating time as a factor. This allows materials to respond to environmental stimuli, such as changes in temperature or pH, adapting to the body’s conditions after implantation. These responsive materials are particularly beneficial in regenerative medicine, where shape-memory polymers or graphene composites integrate seamlessly with native tissues to promote healing and restore function. AI-driven design models simulate the performance of these materials, ensuring robust and long-lasting integration with the body [209]. Nanofiber-based scaffolds, composed of materials like polycaprolactone (PCL) or gelatin, provide a biomimetic environment that supports tissue regeneration. Embedded with bioactive nanoparticles—such as silver or bioactive glass—these scaffolds not only offer antimicrobial properties but also accelerate the healing process [210]. AI systems can optimize scaffold architecture to enhance cellular adhesion and proliferation, significantly improving the efficiency of tissue regeneration and repair [207].

Although these technologies are still in the early phases of research, the combination of AI, ML, and nanotechnology in orthopedics offers significant promise for the future of personalized and precision medicine. Together, they enable the development of highly customized implants that integrate more effectively with biological tissues, reducing the risk of complications and improving long-term surgical outcomes. As research progresses, the ongoing optimization of AI in the design of nanomaterials will unlock even greater potential, poised to redefine the landscape of orthopedic implants and treatments.

## 11. Conclusions

Nanotechnology, heralded as a promising field of advancement, is ushering in a new era in the field of orthopedic research. The commercial successes of nanotechnology underscore its potential as a pivotal factor in clinical practice. Current applications of nanotechnology in orthopedic surgery include drug delivery, implants, tissue engineering, diagnostics, and postoperative infection control. Although the theoretical benefits of nanomedicine, particularly in orthopedics, are beginning to materialize, further analyses are imperative for a comprehensive understanding of safety aspects and the full potential of this innovative technology.

## Figures and Tables

**Table 1:** A summary of the nanostructures approximated in the section, including their modifications and effects.

Nanostructure	Modification	Effect	Source
Gold nanoparticles	Connection with emitting europium and enriched on surfaces rich in calcium ions	Potential in precisely labeling microfractures within bones	[34]
	Connection with contrast agents	Reduction in toxicity of probe	[31]
	Component of biochip	Faster and more precise detection of the osteoporosis-related protein osteoprotegerin	[8]
Nanosensor	Encapsulation molecules capable of detecting NO within biodegradable nanocarriers	Existence of biomarker for degenerative joint diseases, enabling non-invasive and real-time monitoring of their progression	[35]
Nanocrystalline silicone	Addition to flexible nanocomposite	Detection of stress and efficacy of implant monitoring	[36]
Carbon nanotubes	Addition to titanium coatings	Assessment of bone formation because of gauging resistance in implant-developed tissues and discerning conductivity variations	[38,39]

**Table 2:** A summary of the nanostructures approximated in the section, including their modifications and effects.

Nanostructure	Modification	Effect	Source
Gold nanoparticles	None	Enhancement of anti-inflammatory agents by targeting iontophoresis	[40,43]
Diamond nanoparticles	Conjugation with alendronate and nanodiamonds	Bone-targeted delivery system for treating osteoporosis	[46]
	Platform for BMP family protein	Facilitated local accumulation, proliferation, and differentiation of bone cell	[44]
Hydroxyapatite nanoparticles	None	Promotion of osteogenesis	[45]
	Conjugation with simvastatin-loaded poly(N-isopropylacrylamide)	Sustained release of simvastatin to counteract osteoporosis	[45]
Polypeptide and cefazolin nanofilm	None	Reduced infection risks and promoted bone growth	[11,47]
Magnesium nanoparticles	Cooccurrence as a part of biocomposite with collagen hydroxyapatite loaded with cisplatin	Enhancement in efficacy against osteosarcoma cells and reduced side effects	[47,48]
Hyaluronic acid crosslinked with cisplatin	None	Efficient osteosarcoma treatment	[51]
Nanoscale metal–organic frameworks	Connection with calcium zoledronate	Treatment of bone metastases from cancer	[52]
Graphene oxide	Conjugation with folate, polyethylene glycol, green indocyanine photothermal, and photodynamic catalyst	Chemo-photodynamic inhibition of osteosarcoma growth	[52]

**Table 3:** A summary of the nanostructures approximated in the section, including their modifications and effects.

Nanostructure	Modification	Effect	Source
Titanium nanoparticles	None	Enhancement of osseointegration capability and stimulation of new bone development	[77]
Carbon nanotubes	Connection with ultra-high-molecular-weight polyethylene	Enhancement of mechanical reinforcement of the implant	[82]
Tantalum nanoparticles	None	Improvement in biocompatibility and high corrosion resistance	[83,84,85]
Titanium dioxide nanotubes	None	Enhancement of bioactivity and osteointegration of implants	[90]
	Copulin with vancomycin and gentamicin	Improvement of antimicrobial properties	[87,88,89]

**Table 4:** A summary of the nanostructures approximated in the section, including their modifications and effects.

Nanostructure	Modification	Effect	Source
Collagen type I nanoparticles	Collagen–hydrogel nanocomposites	Enhancement of bone mineralization	[67,102]
	Combined with apatite	Promotion of osteoblast proliferation and differentiation	[102]
Magnetic fibrin nanoparticles	None	Enhancement of cell viability and activity	[108]
PLA nanofibers	None	Promotion of cell growth and osteogenic differentiation	[109,110]
Graphene oxide and nanohydroxyapatite	Combined with PLA fibers	Enhancement of biocompatibility, tensile strength, and excellent cell proliferation	[111]
Diamond nanoparticles	None or in combination with PLA/PGLA scaffolds	Enhancement of mechanical and biological properties	[113,114]
Apatite nanorods	None	Enhancement of cellular attachment and mineralization	[116]
Nano-hydroxyapatite	None	Enhancement of formation of new bone	[117]
Calcium phosphate-based nanomaterials	None	Bone regeneration	[121,122]
Cerium oxide nanoparticles	Surface-coated with hydroxyapatite	Promotion of M2 polarization	[138]
Hydroxyapatite nanorods	Cooccurred with chitosan	Reduced macrophage activation while increasing osteoblast proliferation	[92]
Zinc oxide nanoparticles	Cooccurred with microcrystalline bioactive glass scaffolds	Propagation of M1 to M2 macrophage polarization	[139]

**Table 5:** A summary of the nanostructures approximated in the section, including their modifications and effects.

Nanostructure	Modification	Effect	Source
Superparamagnetic iron oxide particles	Cooccurred with cellulose nanocrystals and silk fibroin	Increased regeneration processes	[154]
Nanohydrogel	Combined with cyclodextrins and Fe_3_O_4_	Efficiency of cartilage regeneration under pulsed electromagnetic field stimulation	[157]
Nanostructured collagen–hydroxyapatite complex	Combined with biomimetic osteochondral scaffold	Enhancement and faster regeneration of cartilage	[153]
Nanocomposites of double hydroxide with refined aspartic acid glyceride	Conducted with matrix of PGLA incorporating cartogenin	Enhancement of cell aggregation and improved expression of mesenchymal stem cell cartilage biomarkers	[164]
ECM nanoparticle	None	Promotion of the chondrogenic development of human primary chondrocytes	[168]

**Table 6:** A summary of the nanostructures approximated in the section, including their modifications and effects.

Nanostructure	Modification	Effect	Source
Silver nanoparticles	Amalgamation with polymers	Controlled ion release while minimizing the concomitant risks	[182]
Graphene oxide	None	Increased antibacterial properties, conjoined with antiadhesive and contact properties	[186]
Gold spheres	Loaded antibiotic	Destruction of bacterial films with pulsed laser irradiation	[187,188]
Iron oxide nanoparticles	None	Destruction of bacterial films	[189,190]
Polyurethane membranes	None	Provided an optimal wound environment	[191]

## Data Availability

The data presented in this study are available upon request to the corresponding author.
